# Heparan sulfate proteoglycan triggers focal adhesion kinase signaling during *Trypanosoma cruzi* invasion

**DOI:** 10.1590/0074-02760200143

**Published:** 2020-02-01

**Authors:** Tatiana G Melo, Eveline A Coutinho, Mirian Claudia S Pereira

**Affiliations:** Fundação Oswaldo Cruz-Fiocruz, Instituto Oswaldo Cruz, Laboratório de Ultraestrutura Celular, Rio de Janeiro, RJ, Brasil

**Keywords:** heparan sulfate proteoglycans, focal adhesion kinase, Trypanosoma cruzi, signaling pathway

## Abstract

**BACKGROUND:**

*Trypanosoma cruzi*, the etiologic agent of Chagas disease, is capable of triggering different signaling pathways that modulate its internalisation in mammalian cells. Focal adhesion kinase (FAK), a non-receptor tyrosine kinase protein, has been demonstrated as a mechanism of *T. cruzi* invasion in cardiomyocytes. Since the involved cell surface receptors are not yet known, we evaluated whether heparan sulfate proteoglycans (HSPG), a molecule involved in *T. cruzi* recognition and in the regulation of multiple signaling pathways, are able to trigger the FAK signaling pathway during *T. cruzi* invasion.

**METHODS:**

To investigate the role of HSPG in the regulation of the FAK signaling pathway during trypomastigote entry, we performed heparan sulfate (HS) depletion from the cardiomyocyte surface by treatment with heparinase I or *p*-nitrophenyl-β-D-xylopyranoside (*p*-n-xyloside), which abolishes glycosaminoglycan (GAG) attachment to the proteoglycan core protein. Wild-type (CHO-k1) and GAG-deficient Chinese hamster ovary cells (CHO-745) were also used as an approach to evaluate the participation of the HSPG-FAK signaling pathway. FAK activation (FAK Tyr^397^) and spatial distribution were analysed by immunoblotting and indirect immunofluorescence, respectively.

**FINDINGS:**

HS depletion from the cardiomyocyte surface inhibited FAK activation by *T. cruzi*. Cardiomyocyte treatment with heparinase I or *p*-n-xyloside resulted in 34% and 28% FAK phosphorylation level decreases, respectively. The experiments with the CHO cells corroborated the role of HSPG as a FAK activation mediator. *T. cruzi* infection did not stimulate FAK phosphorylation in CHO-745 cells, leading to a 36% reduction in parasite invasion. FAK inhibition due to the PF573228 treatment also impaired *T. cruzi* entry in CHO-k1 cells.

**MAIN CONCLUSION:**

Jointly, our data demonstrate that HSPG is a key molecule in the FAK signaling pathway activation, regulating *T. cruzi* entry.

Chagas disease, a neglected tropical disease caused by *Trypanosoma cruzi*, is widespread worldwide, due to migration flows from Latin America to other continents,^1^ and is a leading cause of severe cardiomyopathy.^2^ This disease affects 8 million people, mainly in Latin America, and is responsible for 677,000 years of life lost due to disability or death.^3^ *T. cruzi* transmission occurs through vector and non-vector-borne modes (congenital, iatrogenic, transplantation and oral routes), alerting to the need to implement public policies to control this silent disease in non-endemic countries. *T. cruzi* dissemination depends on host colonisation, escape from host defenses, replication and parasite persistence in the vertebrate host.^4^ Mammalian cell entry is a requirement for *T. cruzi* proliferation and spread within the vertebrate host*.* A repertoire of molecules on the surface of mammalian cells, including galectin-3, cytokeratin 18, fibronectin, laminin, heparan sulfate proteoglycan (HSPG), integrin, LDL and bradykinin receptors, modulates *T. cruzi* recognition and entry.^5^ Different *T. cruzi* invasion mechanisms involving IP3-kinase activation, lysosome recruitment (including oligopeptidase B and cruzipain-mediated), sphingomyelinase-mediated membrane repair and autophagic pathways, as well as host cell cytoskeleton mechanisms, have been described.^5^ Many cellular signaling pathways have been reported as driving parasite entrance, including protein kinase activation.^5^

The activation of focal adhesion kinase (FAK), a non-receptor tyrosine kinase, may be subverted by diverse intracellular pathogens,^6^ including *T. cruzi*.^7^ FAK comprises three domains, a kinase domain flanked by two non-catalytic domains, the FERM (ezrin-radixin-moesin) domain at the N-terminus and the focal adhesion targeting (FAT) domain, a kinase domain regulator,^8^ at the C-terminus, which binds to focal adhesion proteins such as paxillin and talin, as well as docking sites for Src homology 3 (SH3) domain-containing proteins.^6^ FAK autophosphorylation at Tyr^397^ (pY^397^) recruits members of the Src family, amplifying FAK activation through the phosphorylation of additional residues (Y576, Y577, Y861 and Y925).^9^

Subversion of the FAK/Src signaling pathway modulates cardiomyocyte infection by *T. cruzi*.^7^ FAK activation, evidenced by FAK Tyr^397^ and c-Src phosphorylation, mediates parasite invasion. FAK inhibition by pharmacological inhibitors (PF573228), silencing by small interfering RNA (siRNA) or reduced expression by tetracycline-uninduced Tet-FAK(WT)-46 cells have been noted to impair *T. cruzi* entry into host cells, supporting FAK signaling involvement as a mechanism of *T. cruzi* invasion.^7^ However, the membrane surface receptor and/or ligands involved in this process have not yet been elucidated.

HSPG and integrin orchestrate FAK signaling pathway activation,^10^ ^,^ ^11^ and both are known to trigger *T. cruzi*-mammalian cell entry.^12^ ^,^ ^13^ Our previous data highlight HSPG, a cell-surface and extracellular matrix molecule composed of repeating hexuronic acid (d-glucuronic acid (GlcA) or l-iduronic acid (IdoA)) and d-glucosamine disaccharide units, as a *T. cruzi* invasion mediator in cardiomyocytes, the most affected cells during human infection.^14^ In addition, we have previously identified *T. cruzi* heparin binding proteins (HBP), presenting 65.8 and 59 kDa molecular masses, that bind to heparin and heparan sulfate (HS).^15^ Thus, we hypothesise that *T. cruzi*-HSPG interaction triggers the FAK signaling pathway, leading to parasite invasion. In the present study, we evaluated the role of HSPG in triggering the FAK signaling pathway during the *T. cruzi* invasion process. Our results demonstrate that HSPG acts as a key molecule in FAK signaling pathway activation, regulating *T. cruzi* entry.

## MATERIALS AND METHODS

*Cell cultures* - Primary cardiac muscle cell cultures were obtained as described previously.^16^ Cardiac ventricles obtained from 18-day-old mouse fetuses were fragmented and enzymatically dissociated with a trypsin (0.025%) and collagenase (0.01%) solution. Isolated cells were cultivated in Dulbecco’s modified Eagle medium (DMEM) containing 7% foetal bovine serum (FBS; Cultilab, São Paulo, Brazil), 2.5 mM CaCl_2_, 1 mM *L*-glutamine (Sigma), 2% chicken embryo extract and 1% penicillin/streptomycin solution (Life Technologies, São Paulo, Brazil) and maintained at 37ºC under a 5% CO_2_ atmosphere. All animal procedures were approved by the IOC Committee of Ethics for the Use of Animals (CEUA license LW15-17).

Chinese hamster ovary (CHO) cell lines, comprising the wild type (CHO-K1) and a mutant type deficient in xylosyltransferase (CHO*-*745), and African green monkey kidney epithelial (Vero) cells were grown in a DMEM/F-12 nutrient mixture medium (GIBCO, Life Technologies, UK) and RPMI 1640 medium (Cultilab), respectively, supplemented with 5-10% FBS, 1.176 g/L sodium bicarbonate, 1 mM *L*-glutamine and antibiotics. At 80-100% confluence, cells were subcultured by dissociation with a trypsin-EDTA solution and maintained in a culture medium at 37ºC in a humidified atmosphere containing 5% CO_2_.

*Parasites and T. cruzi-host cell interaction* - Tissue culture-derived trypomastigotes (TCT) from two distinct *T. cruzi* lineages, Dm28c clone (TcI) and Y strain (TcII), were isolated from *T. cruzi*-infected Vero cells on the 4th day post-infection (dpi). Free trypomastigotes were harvested from the culture supernatant and used to infect the cardiomyocyte cultures and CHO lineages at a ratio of 20 parasites per host cell (20:1).

*Cell treatment* - Cardiomyocytes grown on 60 mm culture dishes (2 x 10^6^ cells) were treated for 24 h at 37ºC with 2.5 mM *p*-nitrophenyl β-D-xylopyranoside (*p*-n-xyloside) or 80 mU/mL heparinase I in serum-free DMEM. Two hours prior infection, the medium was substituted by a fresh medium containing *p*-n-xyloside or heparinase I. Additionally, CHO-k1 (6 x 10^5^ cells) and CHO-745 (8 x 10^5^ cells) were seeded in 60 mm culture dishes in a DMEM/F-12 medium supplemented with 10% FBS. The cells were then infected with *T. cruzi* (Y strain or Dm28c clone) for 1 h at 37ºC. After interaction, free trypomastigotes were removed by washing with phosphate-buffered saline (PBS) and the cultures were processed for immunoblotting assay assessments. Controls consisted of the omission of *p*-n-xyloside and the applied enzyme. All experiments were performed at least three times.

*Cell infection profile* - Isolated cardiomyocytes (1 x 10^5^ cells) and CHO cells, CHO-k1 (1 x 10^5^ cells) and CHO-745 (1.5 x1 0^6^), were plated on 24-well plates containing gelatin-coated glass coverslips. Heparinase I and *p*-n-xyloside-treated and untreated cardiomyocytes and CHO cells (CHO-k1 and CHO-745) were infected with *T. cruzi* (Y strain or Dm28c clone) for 1 h or 2 h at 37ºC. Additionally, CHO-k1 cells were treated for 1 h at 37ºC with 20 µg/mL PF573228, a pharmacological FAK inhibitor, prior to *T. cruzi* interactions. After infection, the cells were fixed for 5 min with Bouin’s solution, stained with Giemsa, dehydrated in an acetone-xylol series and mounted in Permount. The infection level was determined by the random quantification of at least 300 cells using a Zeiss Axioplan microscope. Experiments were carried out at least three times, in duplicate.

*Indirect immunofluorescence* - Uninfected and *T. cruzi*-infected cells, pretreated or not with *p*-n-xyloside or heparinase I, were fixed for 20 min at 4ºC with 4% paraformaldehyde (Sigma Aldrich, São Paulo, Brazil) in PBS. Subsequently, the cells were washed with the blockage buffer [PBS with 4% bovine serum albumin (BSA)] and incubated overnight (4ºC) with the anti-FAK Tyr^397^ primary antibody (1:200, Invitrogen, Frederick, MD). After washing, the cells were incubated with the anti-mouse IgG Alexa Fluor 555 secondary antibody (ThermoFisher). Actin filaments were visualised with AlexaFluor 488-labeled phalloidin (1:1000, ThermoFisher) and DNA was detected with 4’,6-diamidino-2-phenylindole dihydrochloride (DAPI). The analysis was performed using a Zeiss AxioImage M2 microscope with Apotome system.

*Protein extraction and immunoblotting assay* - Cell monolayers were washed with cold PBS (pH 7.4) and lysed with 50 mM Tris-HCl containing 1% Triton X-100 and protease and phosphatase inhibitors. Total protein amounts were determined by the Follin-Lowry method. Protein extracts (20 µg) were resolved by sodium dodecyl sulphate-polyacrylamide gel electrophoresis (SDS-PAGE) and transferred onto nitrocellulose membranes. The membranes were then blocked for 30-60 min in Tris-buffered saline containing 0.1% Tween 20 (TBST) and 5% bovine serum albumin (BSA) (TBST-BSA) before incubation with anti-phosphorylated FAK (1:1000; Invitrogen, Frederick, MD) or anti-FAK antibody (1:500; Santa Cruz Biotechnology). After washing with TBST, the membranes were incubated with horseradish peroxidase (HRP)-conjugated anti-rabbit IgG antibody (1:5000), revealed by chemiluminescence (ThermoFisher) and exposed to X-ray films. Densitometric analyses were performed using the ImageJ software. The anti-glyceraldehyde 3-phosphate dehydrogenase (GAPDH) antibody (Life Technologies) was used as the loading control. The immunoblotting experiments were performed independently three times.

*Statistical analyses* - The Student’s *t*-test was used to determine the significance of differences between the mean values of three assays. A p value ≤ 0.05 was considered significant.

## RESULTS

**Fig. 1:**
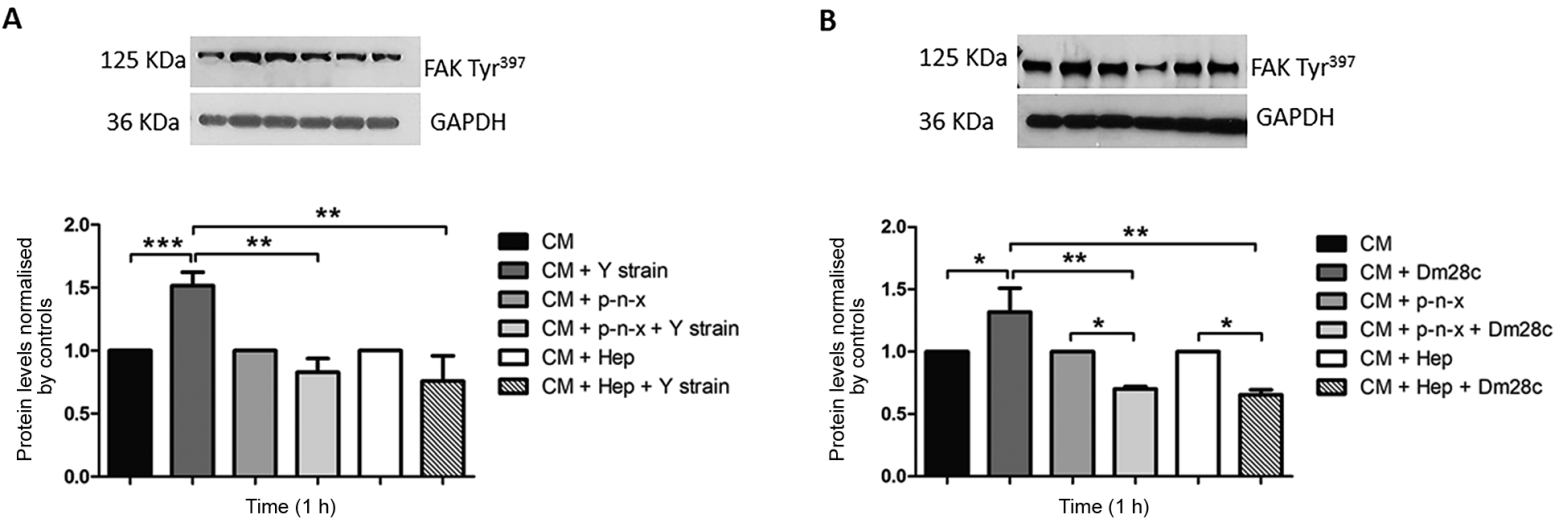
heparan sulfate proteoglycans (HSPG) trigger focal adhesion kinase (FAK) activation during *Trypanosoma cruzi*-cardiomyocyte invasion. Glycosaminoglycans (GAGs) and heparan sulfate (HS) depletion after cardiomyocyte treatment with 2.5 mM *p*-n-xyloside, and heparinase I (80 mU) respectively, prior to Y strain *T. cruzi* infection, inhibits FAK Tyr^397^ up-regulation evidenced in untreated *T. cruzi*-infected cells (A). A western blotting analysis revealed significant increases, of 51% and 31%, in FAK Tyr^397^ phosphorylation levels after Y strain (A) and Dm28c clone (B) interactions with cardiomyocyte, respectively. FAK activation down-regulation, reaching 46% and 50% FAK Tyr^397^ level reductions, was achieved after cardiomyocyte treatment with heparinase I and *p*-n-xyloside, respectively, compared to untreated Dm28c clone *T. cruzi*-infected cardiomyocytes (B). GAPDH was used as the housekeeping control. (*) p ≤ 0.05 and (**) p ≤ 0.004, as determined by the Student’s *t*-test.

**Fig. 2:**
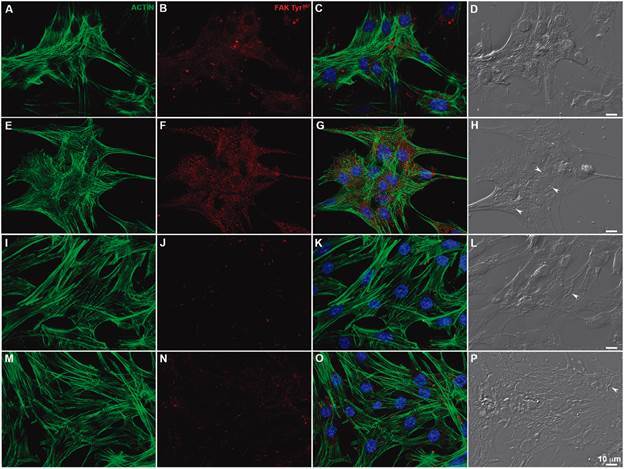
heparan sulfate proteoglycans (HSPG) depletion in cardiomyocytes inhibits focal adhesion kinase (FAK) activation (FAK Tyr^397^) during *Trypanosoma cruzi* invasion. Actin filament (green) and FAK Tyr^397^ (red) distributions in uninfected (A-D) and *T. cruzi*-infected (E-P) cardiomyocytes. Well-developed myofibrils were visualised by phalloidin-Alexa 488 staining (green; A, E, I, M). An intense FFAK Tyr^397^ labeling (red) was noticed in cardiomyocytes after *T. cruzi* (Y strain) interaction (1 h; F) compared to uninfected cells (B). Cardiomyocyte treatment with p-n-xyloside (I-L) or heparinase I (M-P) prior to *T. cruzi* infection (1 h) resulted in FAK activation inhibition, revealed by a weak FAK Y^397^ signal (red; J and N). DAPI, a DNA dye, stained the host cell nucleus and parasite nuclei and kinetoplasts (C, G, K, O). Intracellular parasites (arrowheads) are well-visualised by differential interference contrast (DIC; H, L, P). Merge images (C, G, K and O). Bars = 10 µm.

With the knowledge that HSPG orchestrates *T. cruzi* invasion and also regulates FAK signaling, which is, in turn, involved in *T. cruzi* entry into target cells, we questioned whether *T. cruzi*-HSPG interplay regulates FAK activation during the invasion process. The participation of HSPG as a *T. cruzi* entrance FAK activation regulator was investigated using two *T. cruzi* stocks, the Dm28c clone (TcI) and Y strain (TcII). Two different strategies were employed to determine the role of HSPG in this process, namely HS removal from the surface of cardiomyocytes with heparinase I and proteoglycan synthesis inhibition by *p*-n-xyloside, which abolishes the attachment of glycosaminoglycans to the core protein of proteoglycans by competing for endogenous xylose. As expected, *T. cruzi-*induced the FAK signaling pathway up-regulation in cardiomyocytes, leading to a significant increase in FAK Tyr^397^ phosphorylation levels after 1 h, of 51% and 31% for the Y strain and Dm28c clone, respectively (Fig. 1). Cardiomyocyte treatment with *p*-n-xyloside or heparinase I inhibited FAK signaling pathway activation by *T. cruzi*. HS removal from the surface of cardiomyocytes with heparinase I resulted in a 34% FAK downregulation, whereas the *p*-n-xyloside treatment achieved a 28% reduction in FAK Tyr^397^ phosphorylation levels (Fig. 1) compared to untreated *T. cruzi*-infected cells. Such FAK autophosphorylation (Tyr^397^ residue) inhibition was further accentuated in the interaction with Dm28c clone trypomastigotes, reaching maximum inhibition levels of 46% and 50% after cardiomyocyte treatment with *p*-n-xyloside and heparinase I, respectively (Fig. 1). A reduction in FAK activation was observed only in *T. cruzi* Dm28c-infected cells compared to the phospho-FAK expression in uninfected cultures, suggesting that intrinsic *T. cruzi* genotype characteristics may differentially modulate FAK activation. However, a more detailed analysis is required to clarify this issue. Furthermore, FAK Tyr^397^ distribution was also analysed by immunofluorescence staining. Intense FAK Tyr^397^ labeling was observed throughout the cell cytoplasm, as well as in focal adhesion sites of *T. cruzi*-infected cardiomyocytes compared to uninfected cells (Fig. 2). The fluorescence analyses were consistent with the immunoblotting data, indicating loss of FAK Tyr^397^ signal in infected cardiomyocytes treated with heparinase I or *p*-n-xyloside prior to *T. cruzi* infection (Fig. 2).

**Fig. 3:**
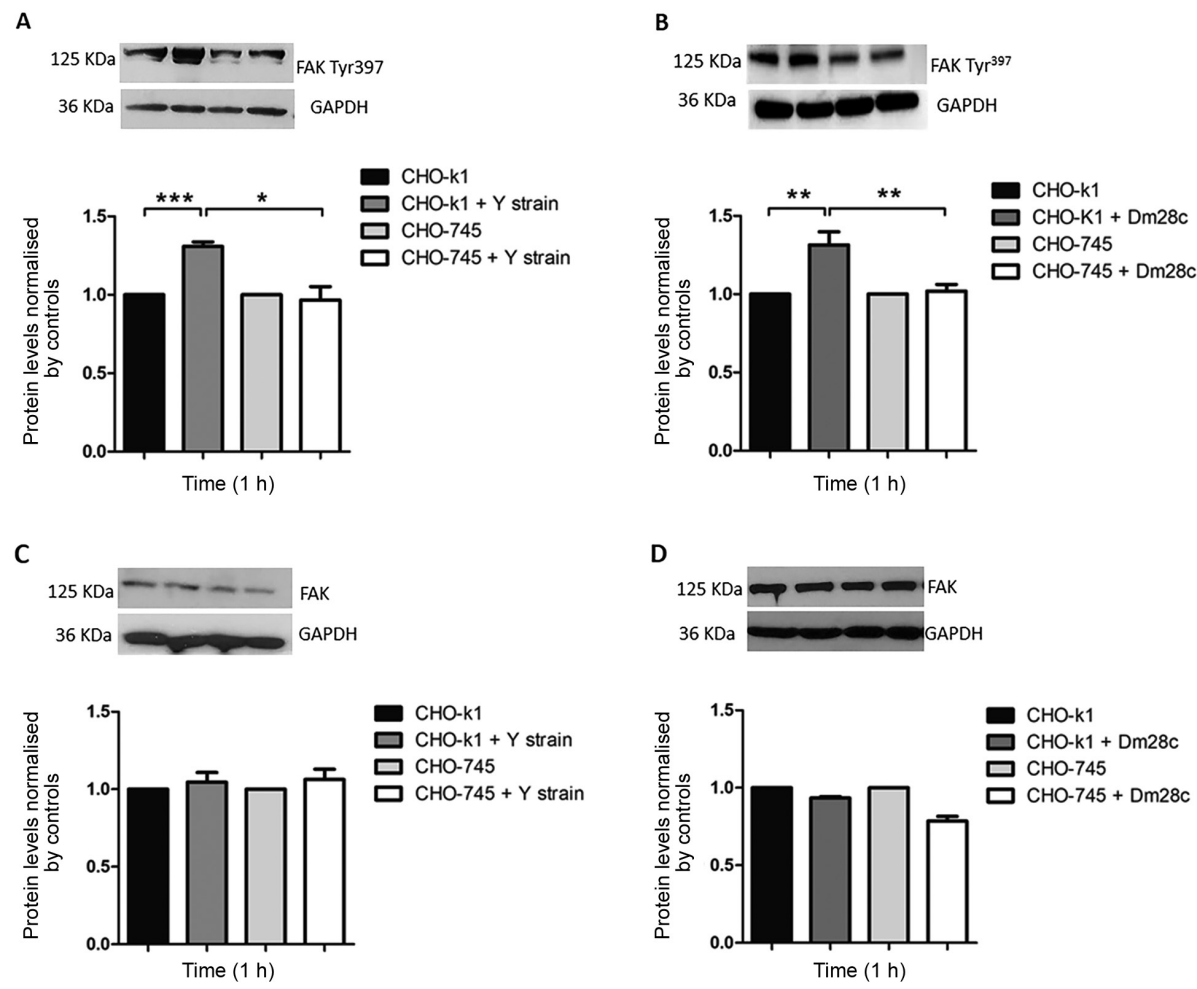
focal adhesion kinase (FAK) signaling modulation in Chinese hamster ovary (CHO) cells invasion by *Trypanosoma cruzi*. Wild-type CHO cells (CHO-K1) and glycosaminoglycan (GAG)-deficient cells (CHO-745) were infected by the *T. cruzi* Y strain (A) and Dm28c clone (B), followed by immunoblotting FAK phosphorylation (FAK Tyr^397^) level detection. Both *T. cruzi* infections (Y strain and Dm28c clone) induced 30% increases in FAK phosphorylation (FAK Tyr^397^) levels in CHO-K1 cells (A and B). In contrast, *T. cruzi*-infected GAG-deficient cells (CHO-745) did not elicit FAK signaling up-regulation (A and B). Total FAK levels were unaltered in CHO cells infected by both *T. cruzi* Y strain (C) and Dm28c clone (D). The anti-glyceraldehyde 3-phosphate dehydrogenase (GAPDH) signal was used to normalise loading differences between the lanes. (*) p ≤ 0.01 and (**) p ≤ 0.005, as determined by the Student’s *t*-test.

**Fig. 4:**
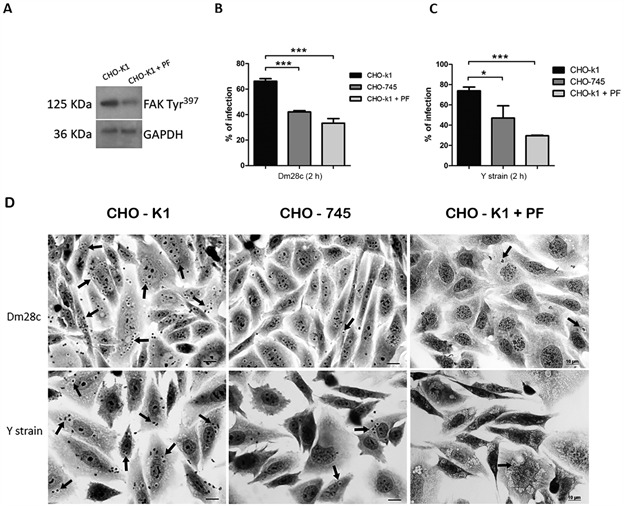
glycosaminoglycans (GAGs) depletion from Chinese hamster ovary (CHO) cell surfaces impaired parasite entry. Wild type CHO-K1 cells displayed 66% and 74% of infection after interaction with *Trypanosoma cruzi* Dm28c clone (A) and Y strain (B), respectively, while infection levels were reduced to 42% and 47% in mutant cells (CHO-745). A 36% inhibition of invasion was evidenced in GAG-deficient cells for both *T. cruzi* stocks. Additionally, treatment of CHO-K1 cells with PF573228 (PF), a pharmacological focal adhesion kinase (FAK) activation inhibitor, impaired parasite entry (B and C). Microscopy images show the *T. cruzi* infection profile in CHO cells. Reduced infection levels and intracellular parasites (arrows; C) are noteworthy in GAG deficient cells (CHO-745) and PF573228 treated CHO-k1 cells. (*) p ≤ 0.02 and (***) p ≤ 0.0002, as determined by the Student’s *t*-test. Bar = 20 µm.

In addition, both CHO lineages were employed to investigate if the HSPG-*T. cruzi* interaction triggers the FAK signaling pathway. The lack of xylosyltransferase activity in CHO-745 cells, leading to glycosaminoglycans (GAG) deficiency, makes this model a suitable tool for HSPG-mediated signaling studies. Subsequently, we evaluated whether HSPG is a key downstream effector of the FAK signaling pathway during invasion by *T. cruzi* of both wild-type and mutant CHO cells. Upregulation of FAK phosphorylation was evidenced in wild-type CHO cells (CHO-K1) infected by *T. cruzi*. The infection by trypomastigotes of both the Y strain and the Dm28c clone of CHO-K1 induced a 30% increase in FAK phosphorylation (FAK Tyr^397^) levels (Fig. 3). In contrast, the infection of CHO 745, glycosaminoglycan-deficient cells, did not stimulate FAK activation. FAK phosphorylation (FAK Tyr^397^) levels remained comparable to those of uninfected CHO-745 cells. Total FAK levels were unaltered in *T. cruzi-*infected CHO-k1 and CHO-745 cells (Fig. 3). Thereafter, we investigated whether FAK activation inhibition was correlated to GAG recognition and *T. cruzi* invasion, assessing both CHO-k1 and CHO-745 infection profiles (Fig. 4). As expected, GAG depletion, mainly HS, from the cell surface impaired parasite entry. Both *T. cruzi* stocks reached 36% of parasite invasion inhibition in GAG-deficient cells. Infection levels were 66% and 74% in wild type cells (CHO-k1) and 42% and 47% in mutant cells (CHO-745) for the Dm28c clone and Y strain, respectively (Fig. 4). As expected, treatment of CHO-k1 cells with the FAK pharmacological inhibitor (PF573228) also reduced parasite entry. Levels of 33% and 29% infection, representing 49.5% and 60% inhibition, were observed for the *T. cruzi* Dm28c clone and Y strain, respectively (Fig. 4). *T. cruzi* invasion impairment is clearly observed in Fig. 4C, highlighting the expressive reduction of intracellular parasites in GAG-deficient cells (CHO-745) and PF573228 treated CHO-k1 cells.

## DISCUSSION

Kinase signaling has been highlighted in the regulation of different biological events involved in target cell-pathogen interactions.^17^ ^,^ ^18^ Activation of signaling pathways during the initial parasite-host cell interface stage promotes *T. cruzi* entry by cytoskeleton-dependent or -independent processes.^5^ FAK, which is involved in cytoskeleton regulation,^19^ is activated during *T. cruzi* cardiomyocyte invasion.^7^ In the present study, we identified HSPG as protagonists in triggering the FAK signaling pathway during *T. cruzi* cell entry.

Parasite surface protein repertoires, capable of triggering different invasion mechanisms, are key to successful target cell entry.^20^ Our data demonstrates that *T. cruzi* strains belonging to different DTUs (TcI and TcII) modulate the FAK signaling pathway. Tissue-derived trypomastigotes (TCT) from the Y strain (TcII) and Dm28c clone (TcI) activate FAK signaling during cardiomyocyte invasion. Interestingly, it has been recently demonstrated that FAK activation is not required for metacyclic trypomastigote (MT) entry into host cells,^21^ showing that FAK depletion increases FAK-deficient HeLa cell susceptibility to MT invasion.^21^ This contrasting finding may be related to the differences between TCT and MT surface molecules involved in host cell invasion. In TCT, proteases, such as cruzipain and oligopeptidase B, as well as active and inactive trans-sialidases, play an important role in this process.^22^ Tc-85, a member of the gp85/trans-sialidase superfamily, presents binding sites for laminin and cytokeratin 18 through the NH2 and COOH terminal domains, respectively, with the latter activating the ERK1/2 signaling pathway during parasite entry. An 85 kDa protein, a TCT ligand that recognises the RGD binding motif in fibronectin, has also been implicated in the invasion process. Gp83 trans-sialidase, a ligand that binds a mammalian cell receptor (p74 receptor), modulates parasite entry by activating host tyrosine kinases in a PKC-dependent MAP kinase pathway.^23^ Integrin, a transmembrane receptor that regulates the extracellular matrix (ECM)-actin cytoskeleton crosstalk, also modulates *T. cruzi* entry. In addition, heparin-binding protein (HBP), a TCT surface molecule that binds heparin and HS, promotes cell invasion in a proteoglycan-dependent manner.^15^ However, their MT surface expression has not yet been elucidated. Evidence has demonstrated that MT entrance relies on lysosome mobilisation, in a gp82- and gp90-dependent manner,^24^ but that lysosome biogenesis and spreading is not essential for TCT invasion.^25^ Additionally, the signaling events triggered by gp35/50, a surface glycoprotein also expressed in MT, modulate cytoskeleton-dependent internalisation,^24^ demonstrating that the glycoprotein profile determines the invasion mechanism process.

FAK activation is known to be modulated by integrin and HSPG on mammalian cell surfaces.^26^ ^,^ ^27^ Regarding the fact that HSPG mediates *T. cruzi* host cell invasion,^13^ ^,^ ^14^ ^,^ ^15^ which seems to be modulated by HBP on the *T. cruzi* surface, HSPG participation as a receptor molecule resulting in downstream FAK activation was also evaluated. As expected, TCT activates the FAK signaling pathway in cardiomyocytes during the invasion process, revealed by FAK phosphorylation. This kinase activation, however, was inhibited, leading to increased resistance to TCT internalisation when HSPG was removed by heparinase I treatment or proteoglycan biosynthesis inhibition by *p*-n-xyloside, demonstrating HSPG involvement in eliciting FAK activation. The lack of a FAK phosphorylation fluorescence signal was also evidenced after GAG and HS removal, supporting the indication that the absence of HSPG down-regulates FAK autophosphorylation (FAK Tyr^397^), decreasing *T. cruzi* uptake by cardiac myocytes. FAK phosphorylation inhibition in GAG-deficient CHO cells, leading to TCT internalisation resistance, also corroborates the fact that HSPG is implicated in FAK activation during *T. cruzi* entry. However, we cannot disregard the integrin role during this process, whose participation in FAK activation has been suggested during *T. cruzi*-cardiac cell interactions^28^ but requires further investigation.

Modulation of the FAK signaling pathway by HSPG interactions has also been reported during Human Papillomavirus Type 16 (HPV16) invasion.^29^ HPV16 binding to HSPG to human transformed keratinocyte (HaCaT cells) surfaces mediates interactions with α6 integrin, regulating FAK activation and the entry of viral particles by filopodia formation. Kaposi’s sarcoma associated herpesvirus (KSHV) binding to human acute monocyticleukemia cell line (THP-1) also involves HS recognition, leading to increased FAK levels, extracellular signal-regulated kinase (ERK1/2) and phosphatidylinositol 3-kinase (IP3K) phosphorylation.^30^ Interestingly, HSPG participation in triggering FAK activation has also been evidenced in non-microbial events. Biological cellular processes in tissue physiology have been demonstrated as regulated by an interstitial flow in a 3-dimensional (3D) microenvironment.^31^ In this scenario, HSPG acts as a mechanosensory molecule, either integrin-dependent or independent, modulating cellular responses to the interstitial flow, leading to FAK activation and downstream ERK signaling, which regulates vascular cell motility.

Together, our data add new insights for a better understanding of FAK activation-mediated *T. cruzi* invasion. *T. cruzi*-HSPG recognition activates the FAK signaling pathway, mediating TCT entry into cardiomyocytes. Since activated FAK regulates members of the Rho GTPase family, including Rac, Cdc42 and RhoA, modulating actin cytoskeleton dynamics, it will be interesting to investigate the complex signaling network involved in this process in detail. A complete understanding of FAK-mediated parasite entry will shed light on *T. cruzi* cardiac cell invasion mechanisms.
